# Somatostatin Analogs Treated Small Intestinal Neuroendocrine Tumor Patients Circulating MicroRNAs

**DOI:** 10.1371/journal.pone.0125553

**Published:** 2015-05-05

**Authors:** Su-Chen Li, Mohid Khan, Martyn Caplin, Tim Meyer, Kjell Öberg, Valeria Giandomenico

**Affiliations:** 1 Department of Medical Sciences, Uppsala University, Uppsala, Sweden; 2 Neuroendocrine Tumor Unit, Centre for Gastroenterology, Royal Free Hospital, London, United Kingdom; 3 The Royal Marsden NHS Foundation Trust, London, United Kingdom; 4 Department of Oncology, UCL Cancer Institute, University College London, London, United Kingdom; 5 Endocrine Oncology Clinic, Uppsala University Hospital, Science for Life Laboratory, Uppsala, Sweden; The University of Hong Kong, CHINA

## Abstract

We previously detected and investigated nine altered microRNAs in small intestinal neuroendocrine tumor (SI-NET) tissues at different stages of disease. The aims of this study are to: 1) analyze whether SI-NET tissue microRNAs can be also detected in patient serum samples, 2) investigate a potential somatostatin analogs (SSAs) role on microRNA levels regulation in SSA-treated patient samples and 3) elucidate whether the serum microRNA levels in samples collected in different hospitals are predictable and steady. Our results show that tissue microRNAs are detectable in patient serum samples, and miR-96, -182, -183, -196a and -200a levels are lower in SI-NET untreated patients than in SSA-treated patients at all different stages. Conversely, miR-31, -129-5p, -133a and -215 levels do not show any difference in untreated SI-NET patients and SSA-treated patients at all different stages. Our findings also show that miR-200a exhibits an atypical behavior with high levels in both untreated and SSA-treated patients at liver metastasis stage, and unequivocally never at the earlier stages. Serum samples collected in two hospitals keep alike microRNA level pattern, elucidating that the results are not dependent on samples handling. In conclusion, SI-NET tissue microRNAs are always detectable in untreated and SSA-treated patient serum samples, SSAs play an unknown role in eliciting SSA-treated patients’ microRNA levels higher than in untreated patients, and this study enlightens that miR-200a might be involved in the liver metastasis during SI-NET progression.

## Introduction

Neuroendocrine tumors (NETs) are a heterogeneous group of malignancies in terms of their biological and clinical features, which cause NETs difficulties in being early diagnosed [1]. NETs are rare malignancies that mainly develop in the gastrointestinal and bronchopulmonary tract [2]. Small intestinal NETs (SI-NETs) are the most common gastrointestinal NETs, and derive from serotonin-producing enterochromaffin cells, which are distributed throughout the human body [3].

The majority of SI-NETs have an indolent course. Surgical resection of primary SI-NETs is the only efficient method for curing patients. However, delayed diagnosis mainly shows that metastases spread to the liver producing inoperable SI-NETs, becoming mainly fatal. Biotherapies using somatostatin analogs (SSAs) are palliative therapies of major clinical importance to reduce symptoms and control tumor growth [4].

MicroRNAs (miRNAs) are short non-coding RNA sequences of 18 to 25 nucleotides involved in a variety of biological processes, such as cell proliferation, differentiation, apoptosis, development and metabolism. They have a role as post-transcriptional regulators, inducing either translational repression of target mRNAs or their target mRNAs degradation [5]. Abnormal miRNA expression in cancer was initially detected in B-cell chronic lymphocytic leukemia using blood samples [6]; however, miRNAs altered regulation has been associated with many solid tumor types as well [7]. Several studies, which include our recent SI-NET miRNA expression profile [8–11], have reported a number of unexpected miRNAs regulation in NETs.

MiRNAs have recently been detected in human body fluids, mainly serum and plasma, and patient blood is easier to collect than tumor tissues [12,13]. Circulating miRNAs have already been used to perform miRNA profiles, and this supports the scientific possibility of miRNAs becoming potential cancer biomarkers [14–17]. Nevertheless, challenges are involved in circulating miRNA detection, such as biofluids contain a lower amount of miRNAs, having more inhibitors of reverse transcriptase than tissues, and there is lack of reliable method to quantitate miRNAs and normalize quantitative real time PCR (QRT-PCR) analysis using serum and plasma samples [18,19]. To outdistance these challenges, adding carrier RNA, such as bacteriophage MS2, during RNA isolation procedure, including synthetic spike-in RNA to monitor sample quality and selecting miR-16 as reliable reference for normalization of QRT-PCR analysis are mainly the best systems to use [20].

The importance of cancer metabolism became an important hall mark [21]. Cancer-relevant pathways and metabolic phenotype of tumors newly addressed the importance of the Warburg effect [22] throughout molecular aspects and therapeutic possibilities. Although normal cells undergo glycolysis and oxidative phosphorylation in the presence of oxygen, proliferating and cancer cells exhibit an increased uptake of glucose and increased rate of glycolysis, predominantly undergo lactic acid fermentation. Today, it is a challenge to understand whether this phenomenon is either the consequence of altered genetic regulation in cancer or it is one cause of cancer development. Hence, it is of importance to remember that miRNAs regulate the aerobic glycolysis through a variety of established mechanisms and pathways as properly described [23]. Because altered regulated miRNAs has already turned out being correlated with diagnosis, staging, progression, prognosis and response to clinical therapies in several malignancies [24], establishing the miRNA roles in tumor biology and cancer metabolism might be important for SI-NETs.

We recently provided a genome-wide miRNA profiling by using SI-NET tissue specimens. Bioinformatics and experimental analyses showed that five tissue miRNAs (miR-96, -182, -183, -196a and -200a) were upregulated, whereas four (miR-31, -129-5p, -133a and -215) were downregulated at all different stages [8]. We first extended our analyses from tissue specimens to serum samples to show that biological fluids are ideal for monitoring SI-NET patients at different stages. We also investigated the nine tissue miRNAs in patient serum samples at different stages, with two other major aims. The second one focused on whether SSAs might play a control role on serum miRNA levels. The third one investigated serum miRNA level patterns, using samples collected in different hospitals, to determine whether the patterns were not dependent on sample handling.

## Materials and Methods

### Serum Samples

A total of 70 serum samples were collected at Uppsala University Hospital (UU) and University College of London (UCL), 49 out of 70 were from UU, 21 out of 70 from UCL (Tables 1 and 2). Seven healthy donor (HD) samples and 42 SI-NET patient samples at different stages: 14 primary tumors (PT), 14 lymph node metastases (LNM) and 14 liver metastases (LM), in summary 49 samples collected at UU (Table 1). We included a total of 21 samples collected at UCL, 7 HD serum samples and 14 SI-NET serum samples at the LM stage, collected at UCL (Table 2). A drug regimen, metastatic site and grade unbiased selection to match UCL samples with the ones from UU resulted in nine samples 3 HD and 6 LM, which were used in the study. The nine bolded samples from UCL, matching the bolded ones from UU in Table 1, are shown in Table 2. In conclusion, 58 samples (49 from UU and 9 from UCL) were used in the study upon unbiased selection of matched samples. Permission to collect serum samples at Uppsala University Hospital was approved by the Regional Ethics Committee Uppsala [Dnr 2011/426], whereas permission to collect serum samples at the University College of London was approved by The East London and The City Alpha Research Ethics Committee [09/H0704/44] with written informed consent provided by all the donors. Eligible patients had pathologically confirmed NETs categorized according to their primary site of origin with metastatic disease measurable by Response Evaluation Criteria in Solid Tumors (RECIST).

**Table 1:** Serum samples collected at Uppsala University Hospital.

ID	Sex/Age*	Treatment	Type	Metastatic site	Grade
HD1	F/42				
HD2	F/51				
HD3	M/35				
**HD4**	F/61				
**HD5**	M/59				
**HD6**	M41				
HD7	M/41				
PT1	M/46	Untreated	Primary		G1
PT2	F/66	Untreated	Primary		G1
PT3	M/64	Untreated	Primary		G1
PT4	M/76	Untreated	Primary		G2
PT5	F/59	Untreated	Primary		G1
PT6	M/74	Untreated	Primary		G1
PT7	F/47	Untreated	Primary		G1
PT8	M/85	SSA	Primary		G1
PT9	M/79	SSA	Primary		G1
PT10	M/70	SSA	Primary		G1
PT11	M/60	SSA	Primary		G1
PT12	F/76	SSA	Primary		G1
PT13	M/79	SSA	Primary		G1
PT14	F/68	SSA	Primary		G1
LNM1	M/70	Untreated	Metastases	Lymph Node	G2
LNM2	F/52	Untreated	Metastases	Lymph Node	G1
LNM3	F/76	Untreated	Metastases	Lymph Node	G1
LNM4	F/76	Untreated	Metastases	Lymph Node	G1
LNM5	M/65	Untreated	Metastases	Lymph Node	G1
LNM6	F/65	Untreated	Metastases	Lymph Node	G1
LNM7	M/64	Untreated	Metastases	Lymph Node	G1
LNM8	F/66	SSA	Metastases	Lymph Node	G1
LNM9	M/75	SSA	Metastases	Lymph Node	G1
LNM10	F/70	SSA	Metastases	Lymph Node	G1
LNM11	F/72	SSA	Metastases	Lymph Node	G1
LNM12	F/50	SSA	Metastases	Lymph Node	G1
LNM13	M/77	SSA	Metastases	Lymph Node	G1
LNM14	M/62	SSA	Metastases	Lymph Node	G1
LM1	M/46	Untreated	Metastases	Liver	G1
LM2	F/51	Untreated	Metastases	Liver	G1
**LM3**	F/57	Untreated	Metastases	Liver	G1
**LM4**	M/45	Untreated	Metastases	Liver	G1
**LM5**	F/63	Untreated	Metastases	Liver	G1
LM6	F/40	Untreated	Metastases	Liver	G1
LM7	M/43	Untreated	Metastases	Liver	G1
LM8	F/70	SSA	Metastases	Liver	G1
LM9	F/71	SSA	Metastases	Liver	G1
LM10	M/57	SSA	Metastases	Liver	G1
**LM11**	M/72	SSA	Metastases	Liver	G2
**LM12**	M/69	SSA	Metastases	Liver	G2
**LM13**	F/51	SSA	Metastases	Liver	G2
LM14	M/65	SSA	Metastases	Liver	G2

Age at the time of sampling (Age*); Healthy donor (HD); Female (F); Male (M); Somatostatin Analogs (SSAs)

Bolded samples were selected to match with UCL samples

**Table 2:** Serum samples collected at University College London, UK.

ID	Sex/Age*	Treatment	Type	Metastatic site	Grade
HD8	M/31				
HD9	M/31				
HD10	F/30				
HD11	M/63				
**HD12**	M/48				
**HD13**	M/55				
**HD14**	F/56				
LM15	F/41	Untreated	Metastases	Liver	G3
LM16	M/83	Untreated	Metastases	Liver	G1
**LM17**	M/55	Untreated	Metastases	Liver	G1
**LM18**	F/66	Untreated	Metastases	Liver	G1
**LM19**	F/65	Untreated	Metastases	Liver	G1
LM20	F/66	Untreated	Metastases	Liver	G2
LM21	M/65	Untreated	Metastases	Liver	G2
LM22	F/75	Untreated	Metastases	Liver	G2
LM23	F/57	Surgery	Metastases	Liver	G1
LM24	F/67	Y90	Metastases	Liver	G1
LM25	F/71	Y90	Metastases	Liver	G2
**LM26**	M/74	SSA	Metastases	Liver	G2
**LM27**	M/69	SSA	Metastases	Liver	G2
**LM28**	F/74	SSA	Metastases	Liver	G2

Age at the time of sampling (Age*); Healthy donor (HD); Female (F); Male (M); Yttrium (Y90); Somatostatin Analogs (SSA)

Bolded samples were selected to match with UU samples

### RNA Extraction

Total RNA containing small RNA, including miRNAs, was isolated by using the mirVana PARIS Kit (Life Technologies, Carlsbad, CA, USA) according to the manufacturer’s instructions, with a few added modifications, as reported in the next paragraph. We added 250 μL serum from each sample to a 1.5 mL RNase-free tube mixed with Denaturing Solution Master Mix, including 2x Denaturing Solution, 1 μg carrier MS2 bacteriophage RNA (Roche Applied Science, Basel, Switzerland) and 25 fmol cel-miR-39 spike-in oligos (Life Technologies). The purified serum RNA was eluted with RNAse-free water (Life Technologies) and was stored at -70°C until further experiments. The RNA concentration was measured by using the NanoDrop 1000 (NanoDrop, Wilmington, DE, USA). The efficiency of RNA recovery among the samples was evaluated by measuring cel-miR-39 expression, by using quantitative real-time PCR (QRT-PCR) analysis.

### Reverse transcription (RT) and Pre-amplification of RT Products

Total RNA (5 μL) was reverse transcribed to cDNA using the TaqMan miRNA Reverse Transcription (RT) Kit (Life Technologies) and miRNA specific stem-loop primers (Life Technologies), according to the manufacturer’s instructions. Pre-amplification was performed following the RT process to increase serum miRNA quantity. Each pre-amplified reaction was carried out in 20 μL comprising 13 μL Platinum PCR SuperMix (Life Technologies), 5 μL 0.2x TaqMan Small RNA Assay (Life Technologies) and 2 μL RT products. The reaction mixture was incubated in the FlexCycler instrument (Analytik Jean AG, Jean, Germany) at 94°C for 2 minutes, and then at 94°C for 15 seconds and 60°C for 1 minute for 15 cycles.

### Quantitative Real-Time PCR

The QRT-PCR reaction was run on the Stratagene Mx3005P real-time PCR System (Agilent Technologies, Santa Clara, CA, USA). Each QRT-PCR reaction was carried out in 20 μL comprising 2x TaqMan Universal PCR Master Mix II without UNG (Life Technologies), 20x TaqMan Small RNA Assay (1 μL) and 10x diluted pre-amplified RT product (5 μL). The reaction mixture was incubated for 2 minutes at 50°C and 10 minutes at 95°C. Then, 40 cycles of 15 seconds at 94°C, and 1 minute at 60°C, were run. The relative miRNA expression was evaluated by using the comparative cycle threshold method (2^-ΔΔCT^) [25] and hsa-miR-16, set to 1, as an endogenous control [20].

### Statistical analysis

The statistical significance of the difference among groups was evaluated by using a one-way ANOVA test followed by either Dunnett’s test or the Bonferroni test, using GraphPad Prism 5 (Graph Pad, Software, La Jolla CA, USA); *p* value < 0.05 was considered significant.

## Results

### QRT-PCR analyses of serum samples from untreated SI-NET patients

**Fig 1:**
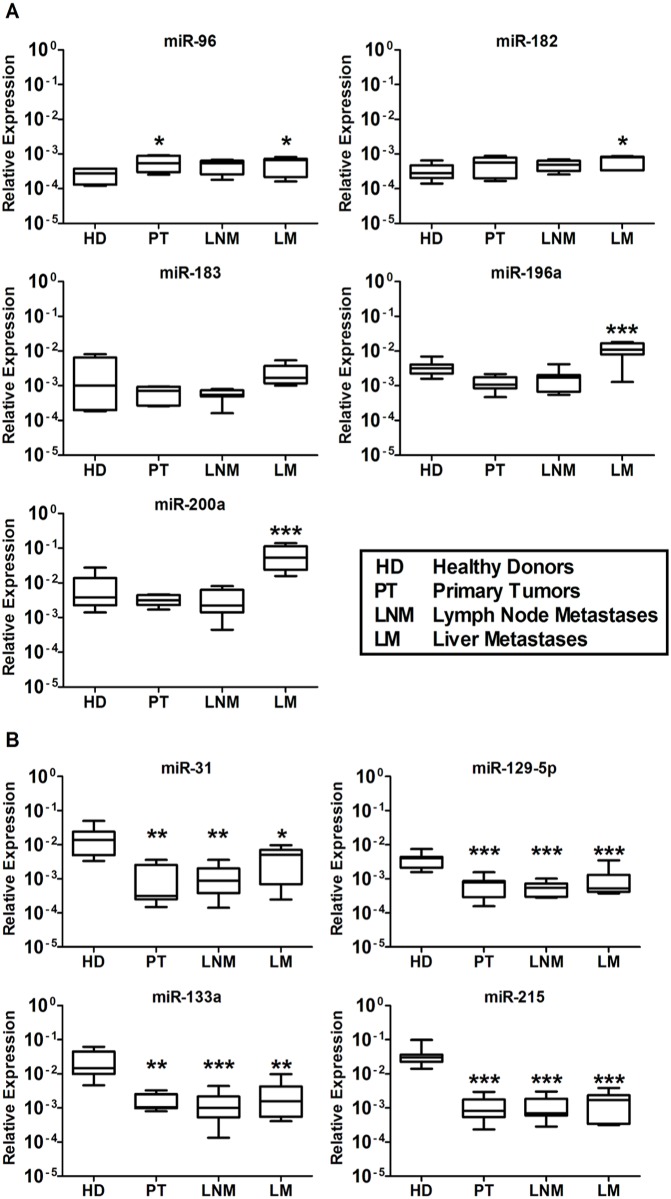
QRT-PCR analysis of untreated SI-NETs at different stages of disease to detect previously analyzed SI-NET tissue miRNAs. Total RNA was isolated from serum samples of seven healthy donors (HD 1–7), seven primary SI-NET (PT 1–7), seven lymph node metastases (LNM 1–7) and seven liver metastases (LM 1–7). (A) MiR-96 was significantly higher in PT and LM patients vs HD, whereas miR-182, -196a and -200a levels were exclusively and significantly higher in LM patients vs HD. Conversely, miR-183 level was never significantly higher in any SI-NET patient group vs HD (B) MiR-31, -129-5p, -133a and -215 levels were significantly lower in untreated patients at different stages vs HD. Results were plotted using the 2^-ΔΔCt^ method with miR-16 expression (set to 1) from each individual sample for normalization. Significance was calculated by One-Way ANOVA followed by Dunnett’s test. * = *p* < 0.05, ** = *p* < 0.01 and *** = *p* < 0.001.

The nine tissue miRNAs were detected from serum samples of seven HD and 21 untreated SI-NET patients (7 PT, 7 LNM, and 7 LM), using TaqMan miRNA array (Life Technologies). The level of miR-96 was significantly higher at the stage of PT and LM, whereas miR-182, -196a and -200a levels were exclusively and significantly higher in LM patients versus (vs) HD. Oddly, miR-183 level was never significantly higher in any SI-NET patient group vs HD (Fig 1A). The level of miR-31, -129-5p, -133a and -215 was significantly lower in SI-NET patients at all different stages vs HD (Fig 1B).

### QRT-PCR analyses of serum samples from SSA-treated SI-NET patients

**Fig 2:**
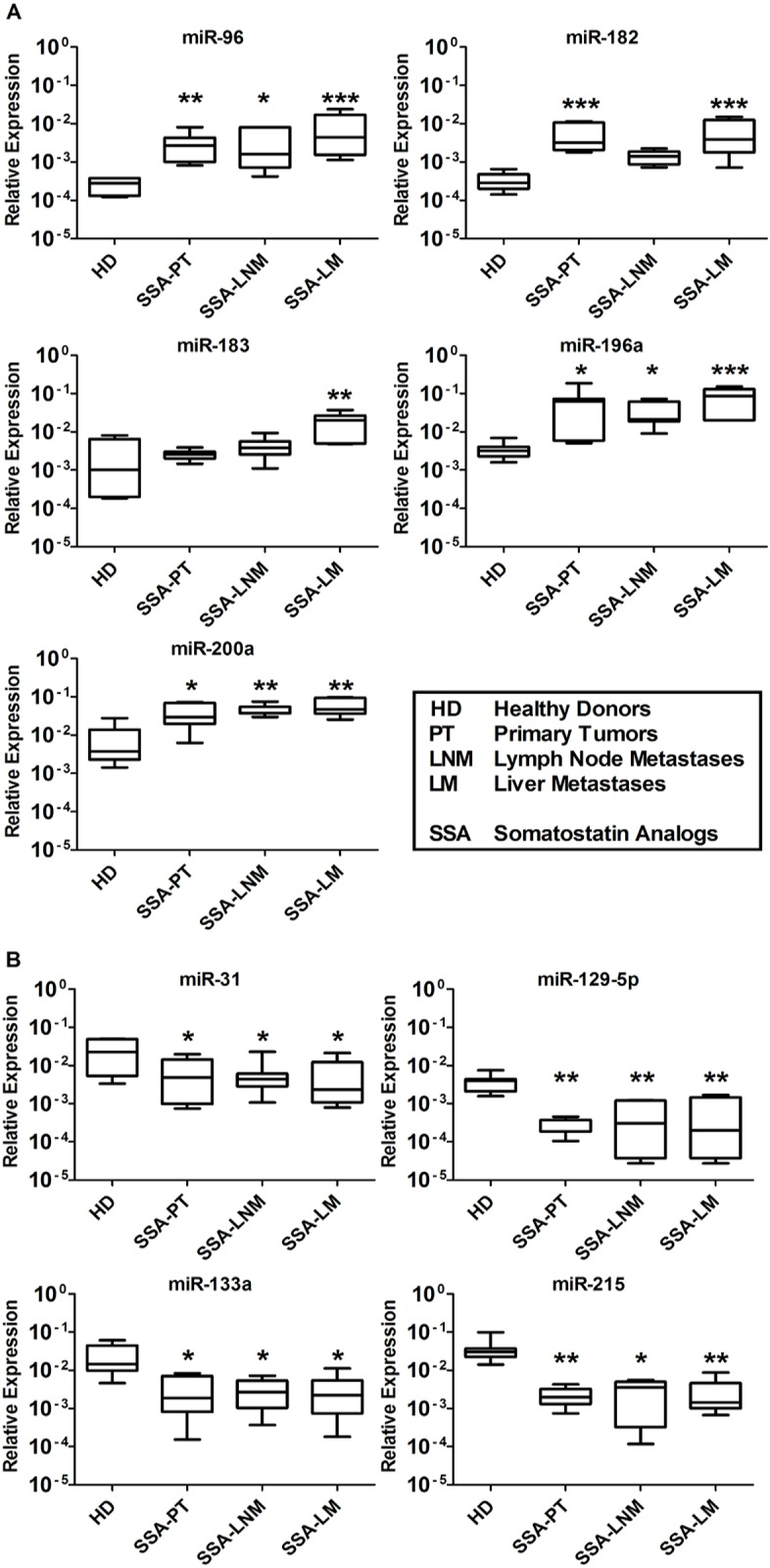
QRT-PCR analysis of SSA-treated SI-NETs at different stages of disease to detect previously analyzed SI-NET tissue miRNAs. Total RNA was isolated from serum samples of seven healthy donors (HD 1–7), seven primary SI-NET (PT 8–14), seven lymph node metastases (LNM 8–14) and seven liver metastases (LM 8–14). (A) MiR-96, -182, -183, -196a and -200a levels were significantly higher at all stages of SSA-treated patients vs HD. (B) MiR-31, -129-5p, -133a and -215 levels were significantly lower at all different stages of SSA-treated patients vs HD. Results were plotted using the 2^-ΔΔCt^ method with miR-16 expression (set to 1) from each individual sample for normalization. Significance was calculated by One-Way ANOVA followed by Dunnett’s test. * = *p* < 0.05, ** = *p* < 0.01 and *** = *p* < 0.001.

The nine tissue miRNAs were clearly detected in serum samples of seven HD and 21 SSA-treated SI-NET patients at different stages (7 SSA-PT, 7 SSA-LNM, and 7 SSA-LM). The level of miR-96, -182, -183, -196a and -200a was significantly higher in SSA-treated patients at all different stages vs HD (Fig 2A). Conversely, the level of miR-31, -129-5p, -133a and -215 was significantly lower in SSA-treated patients at all different stages vs HD (Fig 2B).

### MiRNA expression analysis of untreated and SSA-treated SI-NET patient serum

**Fig 3:**
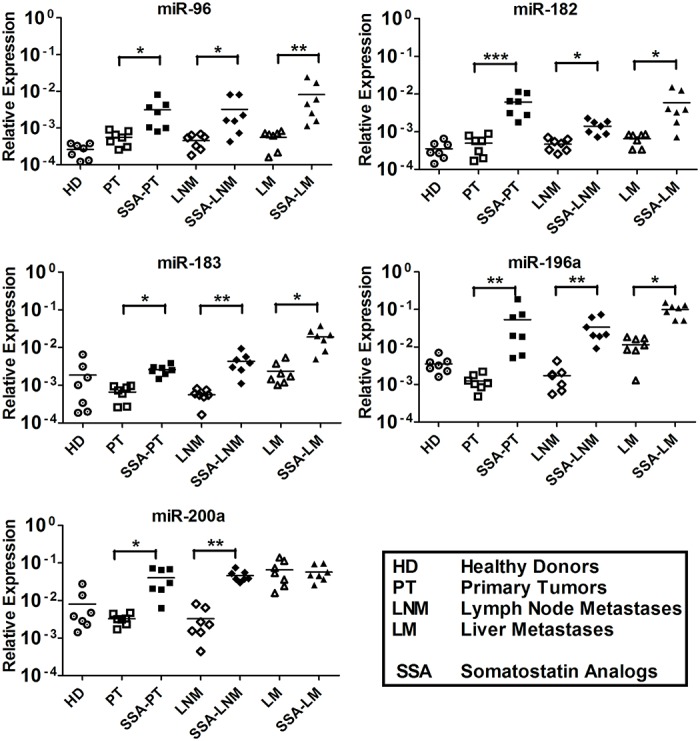
Five upregulated tissue miRNAs were detected in serum samples of healthy donors, untreated and SSA-treated SI-NET patients at different stages. MiR-96, -182, -183, -196a and -200a levels were significantly higher at all stages of SSA-treated patients vs untreated patients. Remarkably, miR-200a showed an atypical behavior because its level was higher in both untreated LM patients and SSA-treated LM patients. The QRT-PCR analysis included seven healthy donors (HD 1–7), 21 untreated patients (PT 1–7, LNM 1–7 and LM 1–7) and 21 SSA-treated patients (SSA-PT 8–14, SSA-LNM 8–14 and SSA-LM 8–14). Results were plotted using the 2^-ΔΔCt^ method with miR-16 expression (set to 1) from each individual sample for normalization. Significance was calculated by One-Way ANOVA followed by the Dunnett’s test. * = *p* < 0.05, ** = *p* < 0.01 and *** = *p* < 0.001.

**Fig 4:**
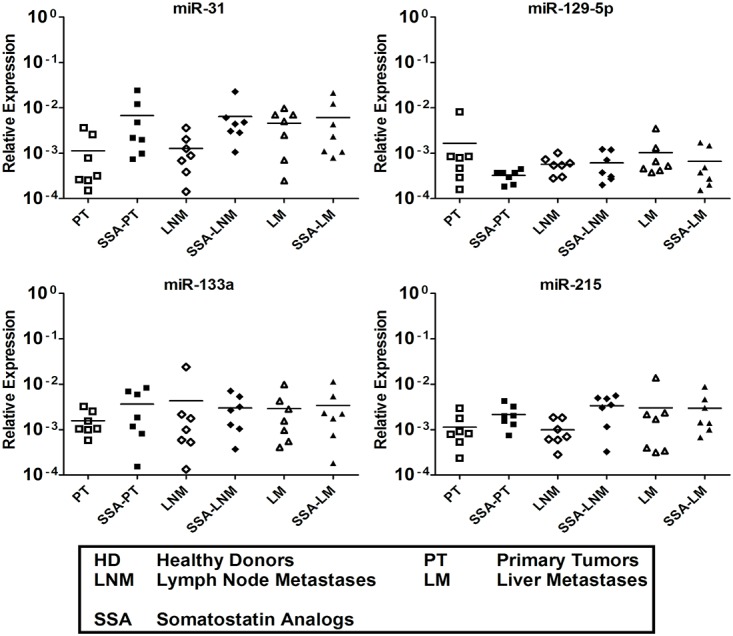
Four downregulated tissue miRNAs were detected in serum samples of untreated and SSA-treated SI-NET patients at all stages. MiR-31, -129-5p, -133a and -215 levels did not show any different expression at all different stages of SSA-treated SI-NET patients vs untreated patients. The QRT-PCR analysis included 21 untreated patients (PT 1–7, LNM 1–7 and LM 1–7) and 21 SSA-treated patients (SSA-PT 8–14, SSA-LNM 8–14 and SSA-LM 8–14). Results were plotted using the 2^-ΔΔCt^ method with miR-16 expression (set to 1) from each individual sample for normalization. Significance was calculated by One-Way ANOVA followed by the Dunnett’s test. * = *p* < 0.05, ** = *p* < 0.01 and *** = *p* < 0.001.

We further explored the nine serum miRNAs level in untreated and SSA-treated SI-NET patients. This investigation aimed at evaluating whether SSAs might have played any role in the miRNA level regulation. QRT-PCR analysis results show that the level of miR-96, -182, -183, -196a and -200a was higher in SSA-treated patients vs untreated SI-NET patients at all different stages. Oddly, miR-200a shows a different level pattern with no significant difference between untreated LM patients and SSA-treated LM patients (Fig 3). Instead, the four miRNAs, miR-31, -129-5p, -133a and -215, characterized by low levels in serum always show a similar level pattern in untreated SI-NET patients and in SSA-treated patients at all different stages (Fig 4).

### Serum miRNA level patterns are alike in samples collected at UU and UCL

**Fig 5:**
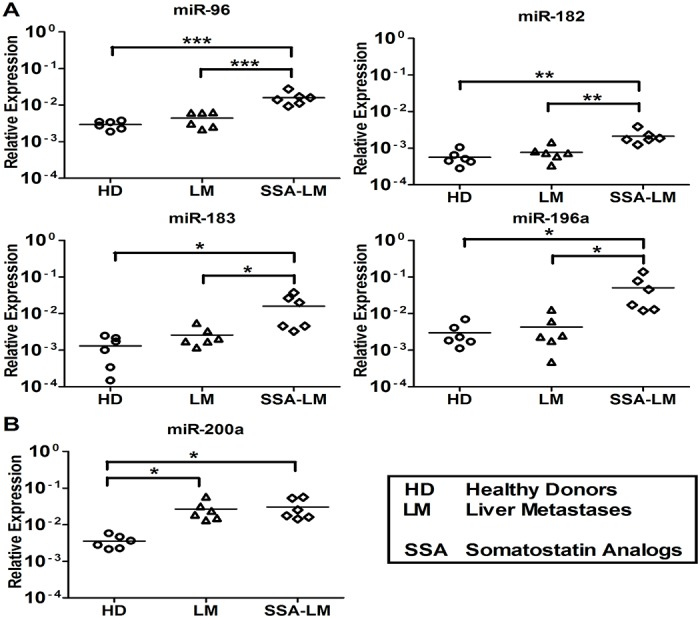
Five upregulated tissue miRNAs were detected in untreated and SSA-treated SI-NET patients at LM stage in serum samples collected at UU and UCL. Total RNA was isolated from UU collection, including three healthy donors (HD 4–6), three untreated liver metastases (LM 3–5) and three SSA-treated liver metastases (SSA-LM 11–13). Total RNA was also isolated from UCL collection, including three healthy donors (HD 12–14), three untreated liver metastases (LM 17–19) and three SSA-treated liver metastases (SSA-LM 26–28). (A) MiR-96, -182, -183 and -196a levels were significantly higher in 6 SSA-treated LM patients vs 6 untreated LM patients and 6 HD. (B) MiR-200a showed the same atypical behavior previously shown in Fig 3. Results were plotted using the 2^-ΔΔCt^ method with miR-16 expression (set to 1) from each individual sample for normalization. Significance was calculated by One-Way ANOVA followed by the Bonferroni test. * = *p* < 0.05, ** = *p* < 0.01 and *** = *p* < 0.001.

**Fig 6:**
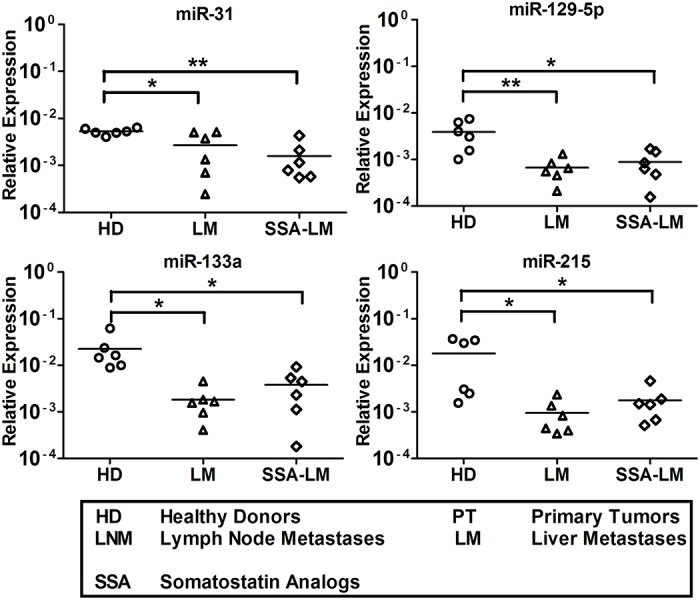
Four downregulated tissue miRNAs were detected in untreated and SSA-treated SI-NET patients at LM stage in serum samples collected at UU and UCL. Total RNA was isolated from UU collection, including three healthy donors (HD 4–6), three untreated liver metastases (LM 3–5) and three SSA-treated liver metastases (SSA-LM 11–13). Total RNA was also isolated from UCL collection, including three healthy donors (HD 12–14), three untreated liver metastases (LM 17–19) and three SSA-treated liver metastases (SSA-LM 26–28). MiR-31, -129-5p, -133a and -215 levels were significantly lower in 6 untreated LM patients and 6 SSA-treated LM patients vs 6 HD. Results were plotted using the 2^-ΔΔCt^ method with miR-16 expression (set to 1) from each individual sample for normalization. Significance was calculated by One-Way ANOVA followed by the Bonferroni test. * = *p* < 0.05, ** = *p* < 0.01 and *** = *p* < 0.001.

Nine out of 49 serum samples collected at UU, which match drug regimen, metastatic site and grade to nine out of 21 serum samples collected at UCL, were used to run independent experiments (Tables 1 and 2). The level of miR-96, -182, -183 and -196a was significantly higher in six SSA-treated LM patients vs six untreated LM patients and six HD (Fig 5A). Oddly, miR-200a was high level in both untreated LM patients and SSA-treated LM patients, as previously shown in Fig 3 (Fig 5B). Conversely, the level of miR-31, -129-5p, -133a and -215 was significantly lower in both untreated and SSA-treated at LM stage vs HD (Fig 6).

## Discussion

Most SI-NET patients receive a delayed diagnosis. Although early tumor detection might lead to curative surgery highlighting the unmet need for novel SI-NET biomarkers to identify and treat such rare tumors, novel SI-NET late biomarkers are also needed to have better SI-NET patient follow-up, and to develop therapeutic drugs to potentially reprogram the cancer glycolytic metabolism. Over recent decades, a variety of gene regulatory mechanisms have been unveiled. Nevertheless miRNAs, which are post-transcriptional regulators, able to control one third of the entire human genome became of major interest. They are correlated to a number of diseases, such as hematological and solid malignancies [6,26], and have been addressed as pivotal regulators of cancer glycolytic metabolism lately [23].

We identified differentially expressed miRNAs in SI-NET tissue at different stages showing how to develop SI-NET miRNAs as novel biomarkers and therapeutic molecular targets [8]. Scientists have been exploring miRNA expression in biological fluids since 2008 [12,13], because body fluid collection, such as serum and plasma, which are liquid biopsies, is much easier than tissue collection, and is advantageous to study rare diseases, such as SI-NETs, to improve prognostics and follow-up markers to improve patient clinical outcome.

Evaluation of tissue SI-NET miRNA in patient serum samples was one of the major aims of this study, to extend the scientific value of our previous study [8]. We thus, first investigated the levels of previously selected tissue miRNAs by using SI-NET patient serum samples. The possible detection triggered the interest to investigate whether SSAs, often used to improve the clinical management of SI-NET patients, might have regulating miRNA levels in serum. Results show that the five upregulated tissue miRNAs (miR-96, -182, -183, 196a and -200a), are present at high level in serum samples from SSA-treated patients, whereas the four downregulated tissue miRNAs (miR-31, -129-5p, -133a and -215) at low level in serum samples from SSA-treated patients.

Different miRNA levels in untreated and SSA-treated patient samples were analyzed in serum for the first time, showing a previously unveiled SSAs regulative role on miRNA levels. This might be investigated in patient tumor tissue in a retrospective manner to confirm the importance of these findings next future. Although five out of five miRNAs show high levels in patients’ serum, they always show higher levels when patients’ are under specific drug regimen, i.e. SSAs. Nevertheless, miR-200a showed an oddly phenomenon, showing that the high miR-200a level at the LM stage is not dependent on SSAs, because miR-200a is at high level in the untreated samples as well. Unexpectedly, the four miRNAs at low levels did not change in expression at any stage of disease in the presence of SSAs treatment.

SI-NET samples collected at Uppsala University showed that the SSA drug regimen has an unknown regulatory miRNA level effect in SI-NET patient serum samples. This finding suggested investigating whether the regulatory effect was relevant and not dependent on different hospitals sample handling. This last investigation turned out showing that the results of UU serum samples were alike to the ones using serum samples collected at UCL, characterized by unbiased identical drug regimen, metastatic site and grade. The remarkable consistency level patterns of the UU and UCL serum samples strengthened the relevant importance of SSAs regulatory effect.

One of the most unexpected findings is that miR-200a at the LM stage shows an oddly pattern level compared to the other four miRNAs at high levels included in the analyses both using serum samples collected UU and UCL. The miR-200a expression pattern level is also independently elevated in the absence of SSAs drug regimen in patients at LM stage, as previously shown by the UU samples analysis. Conversely, the four miRNAs with low levels showed no significant difference between untreated and SSA-treated samples in the experiment carried out with the samples collected in UU and UCL, in agreement with our hypothesis.

Metformin is currently the first-line drug treatment for type 2 diabetes. Besides its glucose-lowering effect, there is a growing interest for the actions of this drug as a relevant potential anti-cancer drug [27]. Over the last few years scientific evidences have shown that metformin exerts its cancer effects through miRNAs modulation to reprogram cancer metabolism on many different tumor types, such as breast, lung and pancreatic cancers [28]. Metformin recently started being evaluated for its role in pancreatic adenocarcinomas and neuroendocrine tumors as well [29]. Growing evidences have highlighted the role of miRNAs as pivotal regulators of metabolic process, such as lipid and cholesterol-synthesis. Although alteration in these processes represents important steps in tumor development, they also offer strategic opportunity to block the activity of specific miRNAs by using synthetic antagomir, also known as anti-miRNAs, as we discussed above. Several studies summarize a variety of miRNAs and their more relevant target genes in cancer, which are modulated by metformin, such as in the mentioned references [28,30].

Some of the most important miRNAs involved in anticancer metformin action are the miR-200 family. Several evidences have shown that cancer progression has similarities with the process of epithelial-to-mesenchimal transition (EMT) [31], and miR-200a is pivotal in this process [28]. Park et al., described that the entire miR-200 family, containing five miRNAs: miR-200a/miR-141; miR-200b/miR200c/miR-429, might become a powerful marker determining the epithelial phenotype of cancer cells by targeting the E-cadherin repressors. Although the miR-200 family members target a similar and highly overlapping set of genes, the relevance of miR-200a in controlling this phenomenon is clearly elucidated [32]. Furthermore, Eades et al. established the effects of the miR-200 family on transformation of the normal mammary epithelial cells showing that miR-200a plays a pivotal role in this [33].

Because very little is known on SI-NETs at the stage of liver metastases and their glycolytic metabolism, depicting miR-200a as the most important miRNA of this last study, revealing an unknown role of SSAs in regulating miRNAs levels is opening a new window of investigation, after miR-200a description in a recent published papers [8]. Thus, the unknown role of SSAs in regulating miRNAs levels might elucidate weather miR-200a regulate the SI-NET metastatic process, controlling target genes involved in the cancer glycolytic metabolism reprogramming.

In conclusion, our study shows that using body fluids instead of tumor tissue is possible to follow up SI-NET patients at different stage of diseases because miRNAs can be evaluated by QRT-PCR analysis. This might offer potential novel drug tests, which are less invasive for the patient and less expensive for the hospital. In addition, SSAs drug regimen has an unknown regulative effect on high level miRNAs in SI-NET patients, whereas this capacity is absent in the ones characterized by low level in patients serum. We have also shown that miR-200a is always at higher level during the liver metastasis stage during tumor progression in SI-NET patients, and that SSAs treatment cannot control miR-200a at this stage of disease at all, differently from the other four high levels miRNAs. This is the most novel and challenging finding for miR-200a in serum samples in our opinion, and it requires further studies to elucidate its role in both tumor progression and potentially in glycolytic cancer metabolism.
